# Practical Application of Polysomnography in Infants

**DOI:** 10.3390/jcm14248670

**Published:** 2025-12-07

**Authors:** Kacper Dera, Michał Ciebiera, Filip Dąbrowski, Teresa Jackowska, Norbert Dera

**Affiliations:** 1 Department of Pediatrics, Bielanski Hospital, 01-809 Warsaw, Poland; tjackowska@cmkp.edu.pl; 2 Department of Gynecology, Warsaw Institute of Women’s Health, 00-189 Warsaw, Poland; michal.ciebiera@gmail.com; 3 Second Department of Obstetrics and Gynecology, Center of Postgraduate Medical Education, 00-189 Warsaw, Poland; 4 Department of Gynecology and Gynecological Oncology, Centre of Postgraduate Medical Education CMKP, 01-813 Warsaw, Poland; fil.dabrowski@gmail.com; 5 Department of Pediatrics, Center of Postgraduate Medical Education, 01-813 Warsaw, Poland; 6 Department of Obstetrics, Perinatology and Neonatology, Center of Postgraduate Medical Education, 01-813 Warsaw, Poland; nderrick@interia.pl; 7 Department of Neonatology and Neonatal Pathology, Warsaw Institute of Women’s Health, 00-189 Warsaw, Poland

**Keywords:** polysomnography, obstructive sleep apnea, apnea–hypopnea index, Pierre Robin sequence

## Abstract

This manuscript presents a comprehensive narrative review of the applications of polysomnography in infants. Considering the growing interest in the early identification of sleep-disordered breathing and its impact on the development of the nervous system, this is an exceptionally important and clinically relevant topic. There is a significant need for a proper understanding of the concept of polysomnography, which would enable its appropriate use in both diagnosis and treatment. This issue becomes particularly important given the limited number of scientific reports addressing the neonatal and infant periods. **Objective:** The usefulness of polysomnography during the first year of a child’s life. **Methods:** Between February and August 2025, a review of publications presenting aspects of polysomnography was conducted. Special attention was given to studies concerning the infant period, published between January 2015 and January 2025. The selection was carried out through the PubMed National Library of Medicine search engine, using the following keywords: “polysomnography”, “obstructive sleep apnea”, and “infant.” **Results:** Based on detailed inclusion criteria, 90 out of 1200 publications were qualified for analysis. **Conclusions:** Polysomnography is used both in the diagnostic process and in qualification for surgical treatment. It enables actions aimed at multidisciplinary management that improve patient outcomes while simultaneously reducing factors that worsen prognosis. At the same time, its usefulness in evaluating the therapeutic process and assessing improvement after both noninvasive and invasive interventions should be emphasized.

## 1. Introduction

A polysomnogram (PSG) is a fundamental tool in the clinical evaluation of sleep and remains the gold standard for diagnosing sleep-related breathing disorders (SRBD), including obstructive sleep apnea (OSA) [1,2,3,4]. The apnea–hypopnea index (AHI) is an inherent concept associated with OSA and PSG [5]. It represents the average number of apnea and hypopnea events occurring per hour of sleep [5]. According to the criteria established by the American Academy of Sleep Medicine (AASM), the severity of apnea can be classified as mild (5–15 events/h), moderate (15–30 events/h), and severe (above 30 events/h) [6]. The index is utilized both to determine the severity of OSA and to evaluate treatment outcomes [7]. In patients with the same AHI (like 60), discrepancies in the results of the evaluated parameters regarding the mean duration of both apnea and hypopnea indicate that it is not the right measurement to determine the severity of the disease or to make treatment decisions. Nevertheless, the mean duration of apnea and hypopnea events may be longer in one patient compared to another (12 s vs. 50 s) [5].

In contrast, when considering the classification of OSA severity in children based on the AHI obtained from sleep studies, an acceptable AHI of 1–3 events/h has been established [8]. This range represents the diagnostic cutoff for OSA and is classified as follows: mild OSA when AHI is <5 events/h, moderate OSA when AHI ranges from 5 to 10 events/h, and severe OSA when AHI exceeds 10 events/h [9,10,11,12]. However, these criteria may vary depending on factors such as age, the presence of additional comorbidities, and other polysomnographic variables, including the presence and duration of oxygen desaturation, the degree of hypoventilation, sleep fragmentation, and reduced total sleep time [12,13]. Over the years, several studies were conducted in healthy children to establish normative data [1,14,15,16,17]. Unfortunately, studies utilizing PSG in infancy have demonstrated considerable variability in testing methodologies and have predominantly excluded newborns [18]. Due to the immaturity of respiratory control centers, delays in nerve conduction caused by neuronal immaturity, greater susceptibility of the respiratory tract and chest walls with lower respiratory reserve, newborns are more predisposed to sleep apnea [19,20]. Therefore, it was important to develop reference values for healthy newborns. This approach stemmed from the need to investigate the relationship between infantile apnea and sudden infant death syndrome (SIDS) [21], airway obstruction—particularly in the context of craniofacial and chromosomal abnormalities [22,23,24]—as well as reduced respiratory reserve in chronic lung disease [25].

Accidental asphyxia resulting from an unstable respiratory drive in newborns represents the leading cause of SIDS [26]. The maturation of central and peripheral chemoreception, neurotransmitter regulation, autonomic responses to hypoxia, and genetic predisposition influence the rhythmic neuronal activity of the brainstem, including respiratory movements [27,28]. Additionally, SIDS has been linked to sleeping position, particularly the prone position [29]. The highest incidence of SIDS is observed between 2 and 3 months of age [30,31]. In newborns, links between sleep position, autonomic function, and breathing disorders during sleep are still unclear, which is why efforts were made to explain the relationship [31]. PSG made it possible to demonstrate the susceptibility of newborns to cardiorespiratory events when sleeping in a prone position by observing cardiac arrhythmias (tachycardia) and respiratory instability [31]. There is a scarcity of reports addressing apneas in infants within the first year of life in association with prenatal exposure to tobacco smoke [32,33,34,35,36,37,38].

In the comprehensive assessment of apnea, it would be ideal to combine data from PSG and studies of sleep behavior of infants at home [39]. Nevertheless, collecting information from the home environment is based on interviews with parents and questionnaires, which include subjective observations of parents, not providing objective, direct information about the infant’s sleep [40,41,42]. Several noninvasive methods are available for out-of-hospital sleep recordings in infants, including the physiological monitoring of respiratory variability, electroencephalography, body movements, heart rate (HR), and video recordings [43,44,45,46]. Supporting the development of algorithms classifying sleep status is possible by automating the analysis of records [47,48,49], such as electroencephalography (EEG) [50,51], electrocardiogram (ECG) [52,53], and respiratory signals [45,52,54,55,56], as well as by video-based assessments of these physiological functions through autovideosomnography [46,57].

Alternative techniques for assessing regular changes in human physical and mental activity include actigraphy and bed mattress sensors (BMS) [4,45]. Actigraphy combines the wireless monitoring of the cardiovascular system with the most common technique for measuring infant sleep [58,59,60]. The method involves evaluating sleep and wakefulness by recording the amplitude of movement by a device located around the infant’s ankle. HR and respiratory rate are utilized to distinguish between rapid eye movement (REM) and non-rapid eye movement (NREM) phases during sleep [61]. Conversely, with the help of BMS, sleep scoring may be performed based on body movements and changes in the breathing pattern, which can be reliably recorded [62,63,64]. No sleep classifiers based on BMS are available for infants [65].

In infants, PSG and how it impacts clinical decision making are extremely essential. Therefore, considering the growing number of studies utilizing PSG in this population, an analysis was undertaken to assess the potential benefits of its application in early infancy, with particular emphasis on its clinical relevance in selected disease entities.

## 2. Materials and Methods

Between February and August 2025, we reviewed research papers published from 1 January 2015 to 1 January 2025 concerning children from birth to one year of age who had been treated with PSG. The selection was conducted using the PubMed National Library of Medicine search engine, employing the following keywords: “polysomnography” and “infant”. We applied appropriate criteria to qualify the literature for our review for the sake of the transparency of the analyzed data. The inclusion criteria were as follows: children from birth to one year of age at the time of the PSG study, and research published and available on the PubMed platform from 1 January 2015 to 1 January 2025. Exclusion criteria: studies conducted on children more than 1 year old. We focused on finding information on the use of PSG in children with various clinical conditions, with particular emphasis on sleep and breathing disorders. At the same time, we tried to include available information in the article, taking into account the comparison of PSG values with alternative methods used in the diagnosis of similar clinical conditions. The entire review, conducted in accordance with the narrative review methodology, was based on a subjective selection of relevant components of the literature. Subsequently, in successive stages, the collected data were synthesized into a coherent whole in line with the authors’ interpretation.

## 3. Results

We obtained 1200 results as a result of the verification for the keyword: “polysomnography in infants”. Considering the above criteria, we qualified 90 articles for a detailed study.

1. Studies evaluating polysomnography for sudden infant death syndrome

Among all the articles included in our review, seven focused on the use of PSG to assess the risk of SIDS in infants. Most of these studies were conducted in 2015.

Table 1 presents a summary of seven studies evaluating the risk of SIDS in infants in relation to sleep position, environmental noise, and the presence of parents with newborns during the early postnatal period.

2. Studies evaluating polysomnography for the diagnosis of sleep disorders in various situations

a. Depending on the infant

This section includes 17 articles focusing on the assessment of PSG in the diagnosis and treatment of sleep disorders. Table 2 presents the effectiveness of PSG in diagnosing sleep disorders in correlation with the child’s developmental stage (age) as well as various clinical conditions, taking into account the course of treatment (pharmacotherapy, respiratory therapy, stimulation).

b. Other factors

A total of 49 studies evaluated the use of PSG in diagnosing sleep disorders in selected clinical situations. These included conditions such as Pierre Robin sequence (PRS), infants with Down syndrome (I-DS), laryngomalacia, gastroesophageal reflux (GER), and prematurity. Table 3 presents a summary of 49 studies on the role of PSG in selected clinical conditions, including congenital anomalies, preterm infants, hypoxia, neuromuscular disorders, and functional gastrointestinal disorders. Among these studies, 17 involved children with PRS, 3 with I-DS, 6 with laryngomalacia, 3 with GER, and 10 with sleep-disordered breathing (SDB). PSG was also utilized in seven studies conducted on preterm neonates [including for the assessment of periodic breathing (PB)] and in three studies evaluating the parent–child relationship.

3. Studies evaluating treatment effectiveness

In seven studies, the authors used PSG to assess the effectiveness of treatment with oxygen therapy as well as methylxanthines. Table 4 presents the utility of PSG in evaluating the treatment of respiratory disorders and apnea of prematurity using oxygen and caffeine citrate.

4. Modern methods and devices for monitoring sleep

In the final section, we identified alternative methods to PSG. Table 5 summarizes 10 studies that present modern methods used in the observation of sleep and breathing disorders, such as actigraphy and BMS, which serve as alternatives to PSG.

## 4. Discussion

1. Usefulness of PSG as a diagnostic test

PSG is used in numerous clinical conditions. Its importance may be considered multidirectional, both in terms of the diagnosis of sleep disorders, heart rate variability (HRV), interaction, and psychomotor development between mother and child, as well as the implementation and conduct of diagnostics and therapy.

A study by Mason et al. [72] showed that repeated naps (short sleeping periods) in infants at 9 months of age were beneficial for the child’s development and the process of remembering. At the same time, they showed that the lack of even a single nap, particularly in the morning, adversely influenced the above processes. Otte et al. [73] defined PSG as the gold standard of sleep annotation in infants by using behavior-based analyses to classify sleep data that were collected from healthy infants. However, they emphasized that further studies on a larger population were necessary to validate the implemented method. Nonetheless, according to the data cited, it is important to note that the appropriate development of patterns in the population of healthy infants constitutes the basis for proper PSG analysis when diagnosing abnormal records.

The breathing pattern is characterized by dynamic changes during sleep. Martin et al. [149] found that breathing became regular during NREM sleep and irregular during REM sleep. Satomaa et al. [74] demonstrated local differences in NREM sleep quality, which were mostly attributed to the brain maturation phase and correlated with psychomotor development. Kochiya et al. [75] investigated the dynamic nature of nocturnal HR variability and extracted a parameter called “RA” to determine the regularity of the HR associated with respiration. As a result of the observation, they suspected that higher RA reflected a regular breathing pattern, especially noted during deep sleep. They also showed an association between higher RA and a decrease in HR, suggesting an increase in parasympathetic activity at the time of recording the above changes. They suggested the possibility of using the method in controlling the variability of position and body movements and its potential usefulness in the early diagnosis and prevention of SIDS.

Jmaa et al. [76] used PSG to determine correlations between cardiorespiratory disorders and postimmune inflammatory response in combination with ibuprofen treatment. This study could be an introduction to a discussion in the context of the possible impact of cyclooxygenase inhibition in the humoral response on immunization [150]. Bat-Pitault et al. [77] demonstrated that the intrauterine environment might be the cause of the child’s increased susceptibility to depression and potentially reduce neuroplasticity. They supported their claim by observing changes in sleep structure in the first months of the life of infants born to mothers suffering from depression. A study by Satomaa et al. [78] revealed a difference in the depth of sleep in 1-month-old infants with its simultaneous age-related transformation. They concluded that there was a probable relationship with the maturation of the cerebral cortex.

Joshi et al. [79] indicated the usefulness of PSG in assessing the effectiveness of home-based noninvasive ventilation (NIV) in infants, especially in children with craniofacial diseases. Such infants experience multifactorial OSA in the first year of life and require multidirectional treatment ranging from respiratory support to surgery. The study showed that long-term continuous positive airway pressure (CPAP) therapy in infants significantly improved PSG parameters and was a viable therapeutic option for home-based respiratory support. Similar conclusions were presented by Al-Iede et al. [80], who demonstrated the effectiveness of home-based CPAP in interstitial lung diseases and cardiac disorders. PSG results improved in terms of gas exchange, sleep, and respiratory effort with CPAP.

CPAP is typically initiated at pressures ranging from 4 to 6 cmH2O, with titration up to 10 cmH2O, and is recognized as an effective and well-tolerated therapeutic approach [45,58]. Evidence regarding the long-term use of CPAP/NPPV in infants is primarily derived from relatively small, single-center, retrospective studies, with additional contributions from prospective registries [151,152]. A high-flow nasal cannula (HFNC) may serve as a viable option for young children with persistent moderate-to-severe OSA following surgical intervention, or as a bridging therapy until definitive treatment is administered, as highlighted by the European Respiratory Society (ERS) [153].

Furthermore, in accordance with the 2024 ERS guidelines, the criteria for defining OSA in children under two years of age may differ from those used for older children. An obstructive apnea–hypopnea index (OAHI) exceeding 5 events per hour may be regarded as within the normal range in neonates. This is attributable to the fact that both OSA and central sleep apnea (CSA) generally decline in frequency during infancy in otherwise healthy children, as well as in those presenting with signs of upper airway obstruction [153]. The ERS statement on OSA in young children further emphasizes that the 95th percentile for the central apnea and hypopnea index (CAHI) varies significantly by age. As such, it is recommended that the definition of OSA for children aged 1 to 23 months be primarily based on the OAHI [154]. By 5 months of age, the mean AHI decreases by approximately 75%, with one-third of this reduction attributed to a decline in CAHI [153].

Ruda et al. [81] demonstrated the diagnostic usefulness of PSG in deciding how to treat patients with bilateral vocal fold dysfunction (BVFD). They determined that PSG was 100% effective in OSA diagnosis, but only 50% effective in the case of feeding dysfunction in the above group of patients. Therefore, it was a supportive study, not a conclusive one as regards the method of therapy. Leon-Astudillo et al. [82] sought to demonstrate the benefit of PSG in the diagnosis and evaluation of SDB. However, since SDB is present in all patients with spinal muscular atrophy (SMA) across its various types, and given the poorly understood and variable symptomatology of the disease despite early diagnosis and treatment, the AHI may not serve as the most reliable outcome measure in this pediatric population.

The analysis of the above papers showed the usefulness and great importance of PSG in the diagnosis, prevention, and treatment of apnea and, thus, the impact on the child’s neurodevelopment. Performing PSG at an early stage of the disorder allows for the elimination of threats and facilitates a sufficiently rapid response to improve gas exchange. This contributes to the preservation of well-being, as well as proper metabolic changes (aerobic metabolism), conditioning the proper development of the child [76,77,78,79,80,81,82].

2. The importance of AHI in PSG in infants

Due to the lack of PSG reference values for newborns, Daftary et al. [19] attempted to determine reference values for PSG variables in healthy newborns. The authors conducted the analysis using a standardized set of PSG results and the AASM interpretation criteria. Their observation, based on the use of the AHI, revealed a significant increase in AHI values accompanied by a decrease in sleep efficiency in newborns, compared to healthy children over the age of 1 year. Similar AHI values were observed in comparison with healthy infants. Similar conclusions may be found in a study by Stefanovski et al. [85], who demonstrated higher values of the CAHI and OAHI in newborns compared to infants. Concurrently, it was observed that a significant spontaneous reduction and shift in the type of apnea occurred during the first six months of life, with a predominance of central or obstructive apnea and minimal obstructive apnea. The authors proposed that, at one month of age, an OAHI greater than 18 events per hour or an obstructive apnea index (OAI) exceeding 9 events per hour could be considered abnormal. A central apnea index greater than 9 events per hour surpasses the 95% confidence interval for infants [77,78]. The above research may be used as a reference point in the assessment of OSA in infants.

Kim James et al. [86] studied infants and noted the highest AHI in patients with craniofacial and airway anomalies, followed by neurological abnormalities, while the lowest AHI was observed in cases of respiratory abnormalities. Similar conclusions arise from the paper by Tawfik et al. [87], who noted that laryngomalacia and craniofacial anomalies were highly predictive as regards hospital-based PSG. Meerkov et al. [123] showed a median AHI of 12.3 apnea episodes per hour with a predominance of CSA in children admitted to the neonatal intensive care unit (NICU) with suspected seizures. Similarly, they demonstrated a higher AHI value in infants with myelomeningocele admitted to the NICU compared to the control group (34.2 apnea events/h and 19.3 apnea events/h, respectively), with a predominance of CSA. In neuromuscular disorders, Chiang et al. [155] noted elevated OAHI and CAHI values in infants with SMA in the first 3 months of life. Henderson-Smart et al. [156] noted that 98% of premature apnea disappeared by 40 weeks of postmenstrual age (PMA) in a significant population of premature infants. According to Eichenwald et al. [157] and Martin et al. [158], multifactorial causes related to CSA, OSA or feeding problems should be considered if apnea persists above PMA.

According to the abovementioned papers, it should be assumed that newborns, due to the specificity of the development and maturation of central nervous system (CNS) functions, require further research and the standardization of results comprising AHI. The proper qualification of newborns to the group with normal or impaired functioning and development necessitates the determination of correct AHI values and referring them to the values developed for newborns.

3. The role of PSG in the management of various clinical conditions

A. Pierre Robin sequence

Khayat et al. [89] determined a strong correlation between the desaturation index and OAHI. Anderson et al. [159] and Lam et al. [160] determined the association of the high prevalence of OSA with craniofacial anomalies, with particular emphasis on PRS. NICU observation revealed that the AHI was 50/h in newborns with PRS and airway anomalies. An improvement was noted after the surgical correction of micrognathia and supraglottoplasty (SGP) performed to treat laryngomalacia [161,162,163]. Kirjavainen et al. [90] sought to find a relationship between OSA and PRS and cleft palate while considering sleep positioning. They concluded that OSA in infants depended more on micrognathia than on cleft palate. They observed that the severity of OSA was higher in patients with micrognathia compared to those without. At the same time, the sleeping position on the back was associated with a higher prevalence of OSA compared to the position on the side or the abdomen. Similar findings were reported by Cielo et al. [91], who observed that micrognathia, rather than isolated cleft palate (ICP), was associated with more severe OSA in comparison to the control group. They observed that OSA improved after surgical correction in the majority of infants with micrognathia, whereas in infants with ICP, OSA improved prior to surgical palate repair. The therapeutic issues associated with OSA severity in patients with craniofacial defects with micrognathia were described in a study by Kochhar et al. [92]. The authors presented the use of PSG in patients undergoing mandibular distraction osteogenesis (MDO) and stated that PSG was a tool that might be used to determine the degree of mandibular distraction needed to alleviate obstruction in infants undergoing MDO. A similar report was published by Kanack et al. [93], who assessed the quality of sleep in infants before and after the MDO procedure. They found that neonates with PRS (characterized by micrognathia, glossoptosis, and airway obstruction) demonstrated significantly elevated OAHI/AHI values and markedly reduced oxygen saturation (SpO2) levels compared to children without airway obstruction. They also observed an improvement in the normative values of neonatal sleep following mandibular distraction. Costa et al. [94] and Bangiyev et al. [95] evaluated improvements in PSG parameters in patients undergoing MDO. Bangiyev et al. found a statistically significant improvement in AHI and hypoxia rates during sleep, with particular emphasis on OSA and hypoxia. Improvements associated with a significant reduction in AHI, OAHI, and CAHI in patients with PRS after MDO surgery were also noted by Lee et al. [96]. At the same time, they emphasized that during the observation of the population analyzed with PRS, no spontaneous reduction in AHI, OAHI, or CAHI was found to be associated with the patient’s age.

Regarding the usefulness of PSG in the diagnosis and treatment strategy of patients with PRS, Coutier et al. [97] determined that it was a valuable tool for optimizing OSA treatment, especially at an early stage of development. Due to the natural development in subsequent months of life with a reduction in retrognathia and glossoptosis due to maxillofacial growth and a reduction in GER and/or the use of a nasogastric tube, the treatment process may be optimized with the appropriate use of PSG, depending on the natural improvement of the patient’s condition [154,164,165]. This is related to both the use of a nasogastric tube and the appropriate length and quality of respiratory support implemented [97]. A similar statement regarding the objective analysis and early application of suitable treatment, thanks to PSG, was presented by Zhong et al. [98]. Broucqsault et al. [99] determined the usefulness of PSG in the objective assessment of the results of alternative treatments in the form of tongue-lip adhesion performed in children with PRS to prevent OSA. Albino et al. [100] reported that non-surgical airway treatment was effective in patients demonstrating consistent weight gain and mild to moderate obstruction on PSG, with an AHI of fewer than 20 events per hour. Similarly, Kam et al. [101], due to the noninvasive nature of PSG, and especially AHI, determined their significant usefulness in decision making and measuring the effectiveness of appropriate medical interventions in patients with PRS. 

The results presented in the above publications indicated the usefulness of PSG both in problematic and diagnostic and therapeutic terms in children with PRS. PSG is a valuable method on the basis of which suitable assessment and qualification for both noninvasive and surgical procedures may be made. At the same time, it is possible to maintain control of the treatment and modify the procedure depending on the improvement of the patient’s clinical condition.

B. Down syndrome

I-DS display distinct physical and physiological characteristics that increase their susceptibility to gastrointestinal disorders, metabolic abnormalities, cardiovascular complications, and early feeding difficulties [105]. As in patients with craniofacial and airway anomalies, patients with various concomitant neurological conditions showed an increased incidence of OSA or hypoventilation compared to children without neurological disorders [166]. An increased risk of OSA was observed in patients with neuromuscular diseases and syndromes associated with hypotonia, including Down syndrome [167,168].

Cho et al. [105] established a correlation between dysphagia and OSA in I-DS, indicating that the severity of dysphagia was associated with the severity of the AHI and the OAHI. A similar statement regarding SDB in I-DS is found in another paper by Cho et al. [106], where early PSG assessment was recommended in I-DS. Both moderate and severe OSA and CSA were confirmed, as well as episodes of hypoxia during sleep and hypoventilation in preterm babies, neonates, and infants, particularly in children with DS and hypothyroidism, as a factor aggravating hypotonia, especially in the first year of life. Goffinski et al. [22] pointed out that, in addition to prematurity, OSA, dysphagia, and gastrointestinal disorders (including GERD), Down syndrome should be an additional factor to be considered for eligibility for PSG testing and the expanded diagnosis of congenital heart disease (CHD). The authors tried to find links between individual disorders. It was noted that both the treatment of OSA using CPAP reduced GERD and, conversely, the treatment of GERD reduced the frequency of OSA in patients with concomitant GERD and OSA [22,169,170,171].

The presented data suggest a significant usefulness of PSG in the diagnostic and therapeutic management of children with genetic disorders, particularly those correlating with other clinical conditions. The use of PSG in such a group of patients enables actions aimed at multidisciplinary management, improving the functioning of patients with the simultaneous reduction in factors worsening the prognosis.

C. Laryngomalacia

Laryngomalacia is one of the most common laryngeal abnormalities in infants. It is also associated with a different sleep microarchitecture [107]. Infants with laryngomalacia are characterized by an elevated AHI, mixed apnea index, CAHI, and alterations in sleep architecture, which may result indirectly from frequent awakenings and reduced slow-wave sleep (N3) [107,172]. Therefore, PSG is also applicable in cases of laryngomalacia. Due to the common occurrence of GER and cardiorespiratory disorders, researchers attempted to demonstrate the relationship between the severity of GERD in cases of the coexistence of both conditions.

The efficacy of nocturnal PSG in the diagnostic and therapeutic process in infants with moderate and severe laryngomalacia was indicated by Lajili et al. [108], who identified AHI as a potential predictor of therapeutic decisions and body mass index (BMI), particularly referring to the need for SGP or NIV. Verkest et al. [109] also noted the effectiveness of CPAP and SGP in OSA patients in the course of laryngomalacia. Furthermore, they identified PSG as a valuable and noninvasive tool for evaluating and monitoring laryngomalacia, particularly when used in conjunction with clinical assessment and endoscopic findings. Additionally, Weinsteinet et al. [110] demonstrated that PSG was one of the components in the qualification of patients for a surgical intervention, along with physical examination and endoscopic results.

Research by Cáceres et al. [107], including patients with severe laryngomalacia, showed that the clinical condition might improve after SGP if the infants were diagnosed with severe OSA based on the OAHI. The cyclic alternating pattern (CAP), a marker of sleep instability in children, was identified by the authors as an early indicator of neurodevelopmental impairment. Therefore, the early diagnosis and evaluation of CAP in infants with laryngomalacia may be a preventive factor and reduce the risk of neurodevelopmental disorders. Similar conclusions may be found in research by Cialente et al. [111], who demonstrated that SGP was a safe and effective procedure in the treatment of severe laryngomalacia in infancy.

Due to the problem of the availability and the degree of difficulty performing PSG, Makhout et al. [112] compared the effectiveness of nocturnal pulse oximetry with PSG in diagnosing OSA in infants with laryngomalacia. In their analysis, the authors noted that pulse oximetry demonstrated high sensitivity and positive predictive value (PPV), but was characterized by low specificity and negative predictive value (NPV). Consequently, they concluded that PSG remains the gold standard for diagnosing OSA. However, they also pointed out that few studies have provided reference values for PSG, polygraphy (PG), and nocturnal oximetry parameters in otherwise healthy children aged 2 years or younger [153]. Simultaneously, according to Rotenberg et al., Kao et al., and Watson et al. [173,174,175], pulse oximetry monitoring in children with sleep apnea could help reduce waiting times for PSG. An additional advantage is that home-based measurements facilitate valuable longitudinal monitoring of patient progress and allow for multiple attempts to obtain high-quality data, all at significantly lower costs and shorter waiting times compared to in-laboratory PSG [176].

The presented analysis showed that PSG was the basis for diagnosis and one of the components of the qualification of patients with laryngomalacia for appropriate treatment.

D. Gastroesophageal reflux disease (GERD)

In contrast, Cheyrou-Lagrèze et al. [115] observed that persistent and symptomatic PB may occur in full-term infants. Furthermore, OSA and/or central apnea syndrome (CAS) were detected in the majority of children with GER. The frequency and number of episodes improved following treatment, declined with age, and typically resolved within the first year of life. These findings emphasize the critical need for timely PSG evaluation in the presence of relevant clinical symptoms. Careful interpretation of polysomnographic findings is essential to distinguish PB from OSA and CAS, as well as to determine whether the observed abnormalities are isolated or secondary to an underlying medical condition. Moreover, it is crucial to propose appropriate management and treatment strategies. Unfortunately, it has been found that the severity of the course of GERD combined with cardiorespiratory disorders could not be clearly determined [113,114]. The mechanisms involved in the pathogenesis of GERD are multiple and include

- Motor abnormalities, including reduced resting tone of the lower esophageal sphincter, transient lower esophageal sphincter relaxations, impaired esophageal acid clearance, and delayed gastric emptying;
- Anatomical factors, such as a hiatal hernia;
- Visceral hypersensitivity;
- Impaired mucosal resistance [177].

A variety of research results on the correlation of GER with cardiorespiratory disorders indicate the need for further research. Considering the apnea mechanism induced by vagus nerve stimulation, the findings of Cheyrou-Lagrèze et al. [115] appear to be justified, as the reflux of gastric contents into the esophagus, coupled with simultaneous stimulation of the vagus nerve endings, may serve as a trigger for apnea.

E. Sleep-disordered breathing in various clinical situations

PSG was also used in the assessment of sleep breathing disorders in various clinical situations. PSG proved useful in the study of the dependence of the degree of saturation on the place of residence, taking into account the altitude above sea level.

Analyses taking into account the altitude of 2560 m above sea level and 3200 m above sea level were conducted by Ucrós et al. [116,117]. They showed lower SpO2 values compared to the population living at sea level, with higher PB and CSA. If apnea events were not associated with PB, CSA results were comparable in both populations.

Pellen et al. [118] demonstrated the usefulness of PSG in the process of preparation for surgery and the control of improvement after the treatment of patients with congenital tracheal stenosis (CTS). They determined the usefulness of NIV both before and after the procedure. Shellhaas et al. [119] evaluated the prevalence of SDB in patients with myelomeningocele. They found a negative effect of SDB in the first weeks of life on cognitive development and behavior in infants with myelomeningocele and confirmed the need for further control. Mehta et al. [120] believed that SDB was common in high-risk newborns and was often associated with the co-occurrence of multisystem diseases. They identified the most common conditions concomitant with SDB, i.e., craniofacial abnormalities and respiratory malformations (38%), and genetic syndromes (26%). Hayashi et al. [56] demonstrated the usefulness of PSG in assessing AHI and OAHI reduction during oxygen therapy in children with CSA. Similar conclusions may be found in a study by Singh et al. [122], in which PSG was used for control using NIV. Meerkov et al. [123] emphasized the potential usefulness of PSG in the diagnosis of SDB in newborns at risk of epileptic seizures and in the prognosis of neurodevelopmental disorders in the course of the coincidence of both disorders.

The association of environmental factors with developmental abnormalities—particularly congenital defects and genetic syndromes—and the occurrence of respiratory disorders highlights the important role of PSG in both diagnostic evaluation and therapeutic management. This approach is crucial for preventing complications and assessing developmental outcomes.

4. Importance of PSG in the management of premature newborns

The population of newborns born prematurely (between 22 and 37 weeks of gestation) is a unique group of patients in whom PSG is particularly useful due to the mechanisms governing the breathing process, immaturity, and different reactions under the influence of stimuli acting on receptors. The tendency to long apnea events in preterm infants is associated with a low response to hypoxia and easily collapsing upper airways. Conversely, hypoxia associated with apnea did not seem to trigger a strong stimulus to stimulate breathing [126].

Seppä-Moilanen et al. [126] found that the PSG record was fragmentary in premature infants due to the frequent spontaneous awakenings observed during this period and the repeated lack of awakenings and sleep interruptions during long apnea events or hypoxia. Similarly, Curzi-Dascalova et al. [178] found that most apnea events, especially central ones, caused no physiological changes indicative of awakening. Siriwardhana et al. [127] drew similar conclusions after analyzing the problem of unstable ventilation control with PB based on the estimation of loop gain (LG) in PSG. Decima et al. [128] noted decreasing HR in infants with bradycardia, hypoxia, and reduced brain oxygenation. Similarly, Yee et al. [129] demonstrated that short apnea events, especially PB, which were often undetected and untreated in neonates, were associated with altered autonomic cardiovascular control. At the same time, Henslee et al. [179] suggested that perinatal respiratory complications and prematurity might exert a lasting effect on cardiovascular control. Patural et al. [180] claimed that the assessment of HRV was an important indicator of health status and a factor that might be used in the prediction of a child’s development.

As regards the influence of external factors on maintaining sleep continuity, Llaguno et al. [130] assessed the destabilizing points based on PSG. They determined that the level of noise and lighting during hospitalization contributed to the disturbance of sleep duration in newborns born prematurely. Therefore, protecting newborns from the influence of external factors plays a key role in the process of shaping their development. Measuring skin temperature using PSG to prevent hypothermia may be important in premature infants. Bach et al. [131] attempted to clarify whether distal skin vasodilation observed in adults at bedtime also occurred in preterm newborns. The analysis provided confirmation of the thesis, which was important in the context of the loss of heat in newborns and the need to compensate for it. Therefore, thermal insulation is so important to prevent weight loss in premature infants. Based on PSG and the sleeping position (prone or supine), Fyfe et al. [66] found that cerebral vascular immaturity might be a factor predisposing premature infants to an increased risk of SIDS in the first year of life.

Due to the physiological immaturity of premature newborns with control disorders and, frequently, different reactions to external stimuli that may contribute to developmental abnormalities and the risk of death in a seemingly healthy infant during sleep, the use of PSG seems to be useful on many levels.

5. The usefulness of PSG in assessing treatment, with particular emphasis on caffeine and oxygen therapy

Atalah et al. [136] attempted to determine the effect of caffeine treatment on sleep and wakefulness parameters, as well as neurodevelopment. Their research revealed no differences either in sleep parameters or in the results of the neurodevelopment of premature infants in the analyzed groups of children undergoing caffeine therapy and without caffeine therapy. It was a very valuable observation due to the confirmed role of caffeine used in the case of apnea of prematurity, despite discussions concerning a possible negative effect of caffeine on sleep quality. Schmidt et al. showed the benefits in premature infants in terms of reducing bronchopulmonary dysplasia, severe premature retinopathy, and improving survival rates. In addition, it is one of the few neuroprotective strategies that showed benefits in reducing cerebral palsy, due to the reduced exposure to episodes of hypoxia [181,182]. Similar conclusions were presented by Seppä-Moilanen et al. [137], who demonstrated that caffeine had a pronounced short-term respiratory stimulating effect, without a stimulating effect on the central nervous system or a negative impact on sleep quality. Simultaneously, an increase was noted in the frequency of awakenings in the course of hypoxia. Rossor et al. [138] also reported a beneficial effect of caffeine on the increase in the ventilation response to hypercarbia.

Das et al. [139] demonstrated the effectiveness of low-flow oxygen in alleviating central, OSA, and mixed apnea, as well as improving mean oxygen saturation in infants with OSA. They pointed out possible mechanisms that included the alleviation of hypoxia-induced hypotonia of muscles dilating the upper airway, with positive effects in terms of increased airway pressure with reduced respiratory muscle fatigue and diaphragmatic contractile force. Similarly, Brockbank et al. [140] demonstrated the beneficial effect of oxygen therapy in infants with OSA. They observed a significant reduction in the frequency of OSA and an improvement in oxygenation, without adversely affecting alveolar ventilation. Seppä-Moilanen et al. [141] also presented the beneficial effect of oxygen therapy on the reduction in PB and the number of desaturation episodes without a significant impact on sleep in late preterm infants. The presented results also confirmed the importance of carotid artery overreactivity in the genesis of PB in premature infants. The value of PSG in the individualization of treatment with the mitigation of the effects of hypoxia episodes and qualification to complete the hospitalization may be found in a study by Flores-Fenlon et al. [142]. The authors emphasized the importance of the occurrence of abnormal PSG features in patients with bronchopulmonary dysplasia (BPD).

The above data confirmed the importance of PSG in the qualification and control of apnea treatment and its impact on the elimination of negative assumptions as to the adverse effects of the treatment.

6. The use of PSG in the assessment of parent–child relationships with particular emphasis on the NICU environment

**Figure 1:**
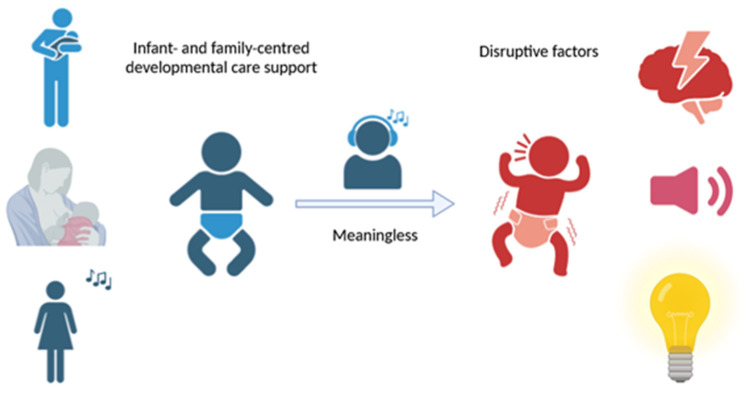
Protective, neutral and stress factors influencing functional and developmental disorders.

An analysis by Shellhaas et al. [133] showed an increased wakefulness of the newborn resulting from hearing the mother’s voice (Figure 1). At the same time, they documented its protective role against potential awakenings caused by a loud sound impulse during children’s hospitalization. Moreover, Levy et al. [134] drew attention to the disturbance of sleep and respiratory disorders associated with various procedures in newborns during their stay at the NICU. Regrettably, the above disorders were observed regardless of the form of the care provided (parent/staff). The authors emphasized the great importance of PSG in the diagnosis of the above conditions due to its much higher sensitivity and specificity compared to standard equipment used in monitoring the well-being of newborns. An interesting statement is also found in an article published by Araújo et al. [135]. The authors demonstrated that hearing protectors used in premature infants to minimize the influence of external stimuli associated with subsequent stress, increased cortisol output, and sleep disorders did not bring the expected effect.

Kacenelenbogen et al. [68] addressed the problem of the presence of both parents in the proper development of an infant. The analysis showed that infants whose parents were separated showed symptoms of delayed development both in terms of psychomotor issues and BMI. They indicated an inseparable relationship of abnormal PSG results with the abovementioned factor.

The presented data indicate a unique stimulus for proper development, i.e., contact with the parent. At the same time, they tackled the issue of the benefits resulting from the presence of both parents. They may also constitute the basis for protective measures in the field of the appropriate management of the newborn in the NICU, while maintaining suitable procedures comprising infant- and family-centered developmental care support.

7. The importance of sleep position in generating apnea

Based on the results presented by Kukkola et al. [69], a higher risk of episodes of apnea and sleep disorders was observed in children with PRS in a supine position and a protective position on the side. Similarly, Wong et al. [29] showed an increased incidence of tachycardia and respiratory instability in the prone sleeping position. They also suggested that, as regards the abovementioned events, it was a more dangerous position compared to the supine one. The results of the analysis by Coutier et al. [70] remained in opposition to the above hypothesis, but they comprised a specific clinical problem, i.e., PRS. They showed an improvement in sleep quality with an incomplete OSA correction in infants with PRS when placed in a prone position. Fyfe et al. [67] demonstrated no significant impact of the prone sleeping position on HR variability in premature infants. Nevertheless, due to the disturbed sympathetic and respiratory balance in the group of extremely preterm infants within 2–3 months of corrected age, they defined the above condition as an increased risk factor for SIDS.

The presented research results indicate the role of PSG in the modification of behaviors and habits, that is, in the prevention of pathological conditions through slight behavioral modifications.

8. Alternatives to PSG as regards methods of diagnosis, assessment of sleep and breathing disorders

Actigraphy is one of the alternative methods most often described in the literature [4,143,144]. Its significance was described by Horger [4], Derbin et al. [143], and Unno et al. [145]. It consists of recording the amplitude of movement in order to distinguish the states of sleep and wakefulness using a sensor placed on the infant’s ankle [4,144]. At the same time, Derbin et al. described actigraphy to be a good sleep index in terms of a wide range of levels of physical activity, but with low specificity regarding the accurate assessment of wakefulness. Therefore, given adequate validation requiring data on both sleep and wakefulness periods, actigraphy, due to the lack of wakefulness periods, is not applicable in the sleep differentiation of preterm infants [143]. Unno et al. [144] emphasized the importance of actigraphy as a method enabling long-term recording of sleep patterns in newborns treated in the NICU at an age equivalent to the date of delivery. The aforementioned factors may play a pivotal role in predicting, diagnosing at an early stage, preventing, and effectively managing sleep disorders, neurological impairments, and cognitive dysfunction.

BMS is another method described by Ranta et al. [45]. It allows the noninvasive control of the breathing pattern and body movements in a long-term manner, especially in patients in the NICU. The authors highlighted its importance, especially in combination with EEG results. A similar conclusion may be found in an article by Moghadam et al. [143]. The authors emphasized the importance of a single EEG channel in controlling changes in sleep phases with a simultaneous clear visualization. The conclusion aligns with the views of Wielek et al. [146] and Koolen et al. [51]. Isler et al. [56] introduced a quantitative approach for classifying active sleep (AS) and quiet sleep (QS) in infants, relying exclusively on measures of respiratory variability. This method was developed to replace the conventional reliance on visual assessment of respiratory variability with an automated approach, achieving reliability rates of 100% for QS and 90% for AS. The method’s validity was confirmed by its high correlation with standard status scores, as well as increased HR variability and elevated high-frequency EEG power during AS compared to QS.

Considering the specificity of infants from PRS, due to the severity of OSA in the group, Coutier et al. [183] pointed out that all-night PG was a valuable alternative to PSG in the diagnosis and assessment of OSA. They also determined that PG showing mild to moderate OSA might constitute a preliminary study, qualifying for expanded diagnostic work-up using PSG of infants with PRS. Bravo et al. [147] identified videonasopharyngoscopy as a viable alternative to PSG for evaluating the severity of SDB in infants with PRS. Conversely, Reddy [23] suggested that infants without significant desaturation events and well-developing ones might only be assessed by oximetry. Conversely, both oximetry and PSG should be performed in infants who are stable in the prone position but have feeding difficulties. As regards patients with desaturation in the prone position, Reddy recommended a nasopharyngeal tube implantation prior to PSG.

The presented data indicate the uniqueness of PSG in diagnostic and therapeutic procedures in special clinical cases in infants. Alternative methods presented in the literature may only serve as preliminary studies used in screening for the qualification for PSG, especially in centers without the possibility of performing such tests. The demonstrated variants are only components presenting selected aspects of the children’s functioning. PSG is the only holistic test showing the correct functioning of the body.

9. Future Ideas

In accordance with the above discussion, it should be emphasized that the neonatal population, due to the specific aspects of development and maturation of CNS functions, requires further research, as well as the standardization and establishment of reference values for the AHI in healthy neonates. At the same time, higher values of CAHI and OAHI in neonates compared to infants indicate that significant spontaneous reduction and a shift in the type of apneas occur during the first six months of life, with a predominance of CSA, obstructive hypopneas, and minimal obstructive apneas. Therefore, when attempting to use behavior-based analyses, further studies on larger populations are necessary to validate the employed method and to develop normative patterns in healthy infants, which constitute the basis for proper PSG analysis in the evaluation of abnormal recordings. Simultaneously, this approach allows for the assessment of positional and body movement variability, with potential utility in early diagnosis and prevention of SIDS.

## 5. Conclusions

The literature data analyzed in this review indicate that the use of PSG is multidirectional. PSG remains the benchmark standard for the diagnosis of OSA, particularly in the context of underlying comorbid conditions. Early implementation of PSG allows for more precise diagnostic evaluation, targeted treatment, and assessment of its impact on neuropsychological development. However, standardized protocols for the infant population, especially for preterm infants, are still lacking. Therefore, further research in this area is warranted.

## Figures and Tables

**Table 1:** PSG for SIDS—summary of selected studies.

No.	Title	Authors	Year of the Study	Study Population	Conclusions
1.	Preterm Infants Exhibit Greater Variability in Cerebrovascular Control than Term Infants [66]	Karinna L Fyfe et al.	2015	52 infants	Cerebrovascular regulation varies between the prone and supine positions in preterm infants.
2.	The Effect of Gestational Age at Birth on Post-Term Maturation of Heart Rate Variability [67]	Karinna L Fyfe et al.	2015	52 infants	Prone sleeping did not significantly affect heart rate variability (HRV) in preterm infants.
3.	Not living with both parents is associated with more health and developmental problems in infants aged 7 to 11 months: a cross-sectional study [68]	Nadine Kacenelenbogen et al.	2015	79,701 infants	This study indicates that not residing with both parents may constitute an independent risk factor for somatic health and psychomotor development in infants.
4.	Obstructive sleep apnea is position-dependent in young infants [69]	Hanna-Leena Kukkola et al.	2023	72 infants	In young infants, obstructive upper airway events are more frequent in the supine position than in lateral sleep positions.
5.	Is prone sleeping dangerous for neonates? Polysomnographic characteristics and NDN gene analysis [29]	Shi-Bing Wong et al.	2019	17 neonates	Tachycardia and respiratory instability observed in neonates during prone sleep suggest an increased susceptibility to cardiopulmonary events in this position.
6.	Impact of prone positioning in infants with Pierre Robin sequence: a polysomnography study [70]	L Coutier et al.	2019	21 infants	Prone positioning of infants resulted in improved sleep quality and only a partial correction of OSA in the vast majority of infants with Pierre Robin sequence (PRS).
7.	Gestational Age at Birth Affects Maturation of Baroreflex Control [71]	Karinna L Fyfe et al.	2015	21 patients	In very preterm infants, maturation of the bioresorbable scaffold was altered after reaching term-equivalent age.

**Table 2:** PSG as a diagnostic test—summary of selected studies.

No.	Title	Authors	Year of the Study	Study Population	Conclusions
1.	The memory benefits of two naps per day during infancy: A pilot investigation [72]	Gina M Mason et al.	2021	15 infants	At 9 months of age, two daily naps, rather than a single nap, are associated with enhanced memory performance.
2.	A behavioral approach to annotating sleep in infants: Building on the classic framework [73]	Renée A Otte et al.	2022	14 infants	Data from a pilot study indicated that the framework produces results comparable to those obtained through annotation based on PSG.
3.	Slow-wave activity and sigma activities are associated with psychomotor development at 8 months of age [74]	Anna-Liisa Satomaa et al.	2020	56 infants	In 8-month-old infants, the quality of NREM sleep exhibited local variations, primarily reflecting the regional phases of brain maturation.
4.	Nocturnal heart rate variability in 1-year-old infants analyzed by using the Least Square Cosine Spectrum Method [75]	Yuko Kochiya et al.	2017	6 infants	In 1-year-old infants, HR regularity fluctuated dynamically overnight.
5.	Cardio-respiratory Events and Inflammatory Response After Primary Immunization in Preterm Infants < 32 Weeks Gestational Age: A Randomized Controlled Study [76]	Wissal Ben Jmaa et al.	2017	362 neonates	In infants born <32 weeks, the first immunization raised C-reactive protein (CRP) levels. Ibuprofen reduced cardio-respiratory events but did not affect CRP or prostaglandin E_2_.
6.	Altered sleep architecture during the first months of life in infants born to depressed mothers [77]	Flora Bat-Pitault et al.	2017	64 infants	Altered sleep patterns were observed during the first months of life in infants born to mothers with depression, suggesting that the prenatal environment may heighten susceptibility to depression and potentially diminish neuroplasticity in these children.
7.	Local changes in computational non-rapid eye movement sleep depth in infants [78]	Anna-Liisa Satomaa et al.	2018	59 infants	Age-related changes in sleep depth are most likely associated with cortical maturation, whereas local variations in sleep depth may additionally reflect the use-dependent properties of slow-wave activity (SWA).
8.	Home continuous positive airway pressure therapy in infants: a single-center experience [79]	Shambhavi Sahotra Joshi et al.	2023	29 infants	Home continuous positive airway pressure (CPAP) is an effective long-term therapy in infancy, with successful weaning possible even after early initiation.
9.	Home continuous positive airway pressure for cardiopulmonary indications in infants and children [80]	Montaha Al-Iede et al.	2018	130 patients	Home CPAP effectively manages interstitial lung diseases and congenital cardiorespiratory disorders, with or without OSA.
10.	Utility of polysomnography and video swallow studies in the management of pediatric patients with congenital idiopathic bilateral vocal fold dysfunction [81]	James Ruda et al.	2020	46 infants	All patients with bilateral vocal fold dysfunction (BVFD) had OSA on postnatal PSG, and ~50% showed feeding dysfunction.
11.	Polysomnography findings in children with spinal muscular atrophy after onasemnogene-abeparvovec [82]	Carmen Leon-Astudillo et al.	2023	8 children	Sleep-disordered breathing (SDB) is prevalent in children with SMA, irrespective of age, treatment status, or level of motor function.
12.	Impact of Infant-Polysomnography Studies on Discharge Management and Outcomes: A 5 Year Experience from a Tertiary Care Unit [83]	Ahmed Fageer Osman et al.	2017	110 neonates	Cardiorespiratory monitoring, medications, and PSG studies do not serve as reliable predictors of outcomes.
13.	Noninvasive Positive Airway Pressure Treatment in Children Less Than 12 Months of Age [84]	Adetayo Adeleye et al.	2016	92 infants	The cohort showed severe SRBD, with treatment often recommended and strong agreement between interpreting and referring physicians.
14.	Polysomnography Reference Values in Healthy Newborns [19]	Ameet S Daftary et al.	2019	30 infants	Newborns showed reduced sleep efficiency and elevated AHI compared to older infants and children, suggesting current sleep apnea severity classifications may not apply to this age group.
15.	Respiratory indices during sleep in healthy infants: A prospective longitudinal study and meta-analysis [85]	Darko Stefanovski et al.	2022	30 infants	Healthy newborns exhibited higher central sleep apnea (CSA) and hypopnea index (CAHI) and obstructive apnea and hypopnea index (OAHI) compared to older children, with a spontaneous reduction in events and shifts in event types observed over the first six months.
16.	Polysomnography use in complex term and preterm infants to facilitate evaluation and management in the neonatal intensive care unit [86]	James Kim et al.	2021	31 infants	The findings indicate that PSG is an essential tool for the assessment and guidance of therapeutic interventions in complex term and preterm infants presenting with a wide range of comorbidities.
17.	Sleep disordered breathing in infants identified through newborn screening with spinal muscular atrophy [87]	Jackie Chiang et al.	2023	11 children	Children treated with onasemnogene-abeparvovec showed decreased SDB over time.

**Table 3:** The role of PSG in the management of selected clinical conditions—a summary of selected studies.

No.	Selected Clinical Conditions	Title	Authors	Year of the Study	Study Population	Conclusions
1.	PRS	Should obstructive hypopneas be included when analyzing sleep studies in infants with Robin Sequence? [88]	Kathleen Lim et al.	2022	20 infants	Incorporating obstructive hypopneas into the evaluation of OSA severity in infants with PRS resulted in a twofold increase in the obstructive event rate.
2.		Polysomnographic findings in infants with Pierre Robin sequence [89]	Abdullah Khayat et al.	2017	46 infants	This study confirmed a high prevalence of OSA in the study population.
3.		Micrognathia and cleft palate as a cause of obstructive sleep apnoea in infants [90]	Turkka Kirjavainen et al.	2025	155 infants	The severity of OSA in infants with PRS is more strongly influenced by the presence of micrognathia than by the presence of a cleft palate.
4.		Evolution of Obstructive Sleep Apnea in Infants with Cleft Palate and Micrognathia [91]	Christopher M Cielo et al.	2016	42 infants	Micrognathia, rather than isolated cleft palate (ICP), was found to be significantly associated with more severe OSA compared to the control group. Both midfacial and mandibular hypoplasia contributed to the presence of OSA in these populations.
5.		Polysomnography-guided mandibular distraction osteogenesis in Pierre Robin sequence patients [92]	Rashi Kochhar et al.	2022	13 infants	This represents the first case series to employ PSG as a guiding tool for mandibular distraction osteogenesis (MDO) in patients with micrognathia, underscoring the need for jaw overcorrection to achieve resolution of OSA.
6.		Normal Neonatal Sleep Defined: Refining Patient Selection and Interpreting Sleep Outcomes for Mandibular Distraction [93]	Melissa D Kanack et al.	2022	13 neonates	“Normal” neonates show more frequent obstructive events and lower oxygen nadirs than previously thought.
7.		The effect of mandibular distraction osteogenesis on airway obstruction and polysomnographic parameters in children with Robin sequence [94]	Amanda Lucas da Costa et al.	2018	38 patients	MDO is an effective surgical intervention in pediatric patients, as evidenced by significant postoperative improvements in clinical symptoms, endoscopic grading assessments, and polysomnographic outcomes.
8.		Sleep architecture in Pierre-Robin sequence: The effect of mandibular distraction osteogenesis [95]	J.N. Bangiyev et al.	2016	32 infants	MDO significantly improved sleep architecture in infants with PRS, reducing obstructive apneas, hypopneas, AHI, OAHI, and several hypoxia indicators during sleep.
9.		Characteristics of sleep apnea in infants with Pierre-Robin sequence: Is there improvement with advancing age? [96]	Jake J. Lee et al.	2015	141 infants	Unlike prior studies in non-PRS patients, no age-related reduction in CSA or OSA severity was seen in infants with PRS.
10.		Role of polysomnography in the management of obstructive sleep apnea during the first year of life in Robin sequence: A prospective and longitudinal study [97]	Laurianne Coutier et al.	2025	45 infants	Sleep and OSA improved spontaneously in PRS infants, nearing normal by 8 months.
11.		[The significance of evaluation of sleep respiration in infants with Pierre Robin sequence] [98]	Jianwen Zhong et al.	2020	17 patients	Most infants with PRS exhibit sleep apnea and hypoxemia, necessitating early intervention and management.
12.		Evaluation of the efficacy of tongue-lip adhesion in Pierre Robin sequence [99]	H. Broucqsault et al.	2018	37 patients	Tongue–lip adhesion effectively alleviated airway obstruction in all infants with PRS and achieved complete resolution of OSA in 29 patients.
13.		Clinical Factors Associated with the Non-Operative Airway Management of Patients with Robin Sequence [100]	Frank P Albino et al.	2016	32 infants	Nonsurgical airway management proved effective in patients who maintained consistent weight gain and exhibited mild to moderate obstruction on PSG, with a mean AHI of fewer than 20 events per hour.
14.		Surgical versus nonsurgical interventions to relieve upper airway obstruction in children with Pierre Robin sequence [101]	Karen Kam et al.	2015	139 patients	In this study, syndromic PRS and low birth weight patients often received early interventions like tracheostomy, with limited use of objective airway measures.
15.		Three-dimensional airways volumetric analysis before and after fast and early mandibular osteodistraction [102]	Valerio Ramieri et al.	2017	4 patients	Fast early mandibular osteodistraction (FEMOD) effectively improves airway function and breathing in PRS and syndromic micrognathia patients. Three-dimensional volume rendering proved valuable for assessing airway volume increases.
16.		Association of polysomnographic parameters with clinical symptoms severity grading in Robin sequence patients: a cohort nested cross-sectional study [103]	Denise Manica et al.	2018	80 patients	Polysomnographic parameters significantly correlated with clinical severity in PRS patients, with oxyhemoglobin saturation measures showing notably high R^2^ values.
17.		Can telemetry data obviate the need for sleep studies in Pierre Robin Sequence? [104]	Nicole Leigh Aaronson et al.	2017	46 infants	In the assessment of infants with PRS, there was a high index of suspicion for OSA. In this series, telemetry data proved insufficient for reliably excluding severe OSA.
18.	I-DS	Dysphagia severity is associated with worse sleep-disordered breathing in infants with Down syndrome [105]	Yeilim Cho et al.	2023	40 infants	Individuals with I-DS exhibited a notably high prevalence of dysphagia and SDB. The severity of dysphagia was found to correlate with the severity of the OAHI.
19.		The burden of sleep disordered breathing in infants with Down syndrome referred to tertiary sleep center [106]	Yeilim Cho et al.	2023	40 infants	This study emphasizes the substantial prevalence of SDB among individuals with I-DS referred to a sleep center, underscoring the necessity of PSG assessment in this patient population.
20.		Obstructive sleep apnea in young infants with Down syndrome evaluated in a Down syndrome specialty clinic [22]	Alida Goffinski et al.	2015	59 infants	OSA is common and often severe in young I-DS infants. Medical issues like gastrointestinal problems, dysphagia, and congenital heart disease (CHD) can help identify those at higher risk.
21.	Laryngomalacia	Characterization of cyclic alternating pattern in infants with laryngomalacia [107]	Laura Mendoza Cáceres et al.	2022	25 infants	Given sleep’s importance in neurodevelopment, clinicians should monitor infants with laryngomalacia for developmental delays, using the cyclic alternating pattern (CAP) as an early indicator to enable timely intervention.
22.		Nap polysomnography in infants with laryngomalacia as a tool to predict treatment strategy [108]	Mariem Lajili et al.	2024	39 infants	AHI from PSG may predict treatment needs and weight gain, supporting its use in diagnosis and management.
23.		Prevalence of obstructive sleep apnea in children with laryngomalacia and value of polysomnography in treatment decisions [109]	Valérie Verkest et al.	2020	64 patients	OSA was diagnosed in 77.4% of laryngomalacia patients and may worsen symptoms, leading to interventions like CPAP or SGP.
24.		Utility of polysomnography in determination of laryngomalacia severity [110]	Jacqueline E Weinstein et al.	2017	25 patients	In this cohort, PSG did not effectively assess laryngomalacia severity or predict surgery need.
25		Changes in Breathing Patterns after Surgery in Severe Laryngomalacia [111]	Fabrizio Cialente et al.	2021	81 infants	Supraglottoplasty (SGP) is a safe, effective treatment for severe laryngomalacia, improving breathing as shown by lung function tests.
26.		Nocturnal pulse oximetry as a possible screening method for obstructive sleep apnea in infants with laryngomalacia [112]	Sanae Makhout et al.	2022	53 patients	Overnight pulse oximetry showed high sensitivity and positive predictive value (PPV) for diagnosing OSA in infants with laryngomalacia, but low specificity and negative predictive value (NPV) mean PSG remains necessary to rule out OSA when oximetry is normal.
27.	GER	Effect of Severity of Esophageal Acidification on Sleep vs. Wake Periods in Infants Presenting with Brief Resolved Unexplained Events [113]	Janani Sankaran et al.	2016	25 infants	Severe esophageal acid exposure (ARI > 7%) was linked to more reflux symptoms when awake, while sleep appeared protective, possibly due to higher chemosensory thresholds.
28.		The Role of Sleep in the Modulation of Gastroesophageal reflux and Symptoms in NICU Neonates [114]	Aslam Qureshi et al.	2015	18 neonates	Contrary to expectations, GER frequency was lower during sleep, but its characteristics and symptom mechanisms differed.
29.		Persistent and symptomatic periodic breathing beyond the neonatal period in full-term infants: A case series [115]	Océane Cheyrou-Lagrèze et al.	2024	10 infants	This study documents instances of persistent, symptomatic PB persisting beyond the first month of life in term-born infants.
30.	SDB	Oxygen saturation, periodic breathing and apnea during sleep in infants 1 to 4 month old living at 2560 m above sea level [116]	Santiago Ucrós et al.	2015	35 infants	Oxygen saturation (SpO2) levels were reduced compared with sea-level values, accompanied by elevated indices of both PB and CSA.
31.		Oxygen saturation, periodic breathing, and sleep apnea in infants aged 1–4 months old living at 3200 m above sea level [117]	Santiago Ucrós et al.	2017	18 infants.	SpO2 was lower than that observed at sea level, whereas both PB and the CSA index were elevated, even after excluding apneas associated with PB.
32.		Use of non-invasive ventilation in children with congenital tracheal stenosis [118]	G. Pellen, C. Pandit et al.	2019	16 patients	Patients with congenital tracheal stenosis (CTS) exhibit obstructive SDB. Trials of noninvasive ventilation (NIV) are generally well tolerated and lead to improvements in SDB.
33.		Sleep-disordered Breathing Among Newborns with Myelomeningocele [119]	Renée A Shellhaas et al.	2017	20 newborns	The results suggest that infants with myelomeningocele could benefit from early, systematic screening for SDB as early as the first week of life.
34.		Clinical characteristics, associated comorbidities and hospital outcomes of neonates with sleep disordered breathing: a retrospective cohort study [120]	Bhavesh Mehta et al.	2024	8 neonates	SDB poses a significant challenge in high-risk neonates, often associated with multiple comorbidities across various organ systems, thus requiring a multidisciplinary approach to optimize management.
35.		Central sleep apnea in otherwise healthy term infants [121]	Ayaka Hayashi et al.	2022	52 infants	CSA in otherwise healthy term infants is typically associated with a favorable prognosis, with oxygen therapy often prescribed for approximately six months.
36.		Polysomnography in infants with clinical suspicion of sleep-related breathing disorders [122]	Jagdev Singh et al.	2022	161 infants	Polysomnographic sleep metrics and the quantity of prescribed treatments were consistent, regardless of whether the PSG was performed during the day or at night.
37.		Sleep-disordered breathing is common among term and near term infants in the NICU [123]	Meera S Meerkov et al.	2019	48 infants	SDB is prevalent in term and near-term newborns at risk for seizures.
38.		Current Practice Patterns in the Diagnosis and Management of Sleep-Disordered Breathing in Infants [124]	Rachana Kombathula et al.	2019	54 infants	The results revealed significant variability in the practice patterns for diagnosing and managing SDB in infants.
39.		Automated detection of sleep apnea in infants: A multi-modal approach [125]	Gregory Cohen et al.	2015	1079 infants	A multimodal approach combining ECG and pulse oximetry improves infant sleep apnea detection over single methods.
40.	Premature newborn	Spontaneous and apnea arousals from sleep in preterm infants [126]	Maija Seppä-Moilanen et al.	2021	21 infants	In preterm infants, frequent spontaneous arousals cause fragmented sleep, but prolonged apneas or hypoxia rarely trigger arousals.
41.		Ventilatory control instability as a predictor of persistent periodic breathing in preterm infants [127]	Leon S Siriwardhana et al.	2022	32 infants	The progression of PB in preterm infants was associated with variations in loop gain (LG).
42.		The longitudinal effects of persistent periodic breathing on cerebral oxygenation in preterm infants [128]	Pauline F F Decima et al.	2015	24 infants	Most preterm infants discharged without clinical respiratory issues had persistent PB.
43.		Autonomic cardiovascular control is altered by intermittent hypoxia in preterm infants [129]	Alicia K. Yee et al.	2023	40 infants	This study presents new evidence that short apneas, especially PB, which are often overlooked or left untreated in neonatal units, can significantly affect autonomic cardiovascular control.
44.		Polysomnography assessment of sleep and wakefulness in premature newborns [130]	Nathalie Sales Llaguno et al.	2015	13 infants.	Preterm newborns spent more time asleep than awake, with quiet sleep constituting the predominant sleep stage.
45.		Distal skin vasodilation in sleep preparedness, and its impact on thermal status in preterm neonates [131]	Véronique Bach et al.	2019	18 neonates	Compensating for body heat loss and maintaining homeothermia would necessitate a 4% increase in metabolic heat production.
46.		The optimization of home oxygen weaning in premature infants trial: Design, rationale, methods, and lessons learned [132]	Alexander Procaskey et al.	2018	196 infants	This trial presents a valuable opportunity to assess a novel home monitoring intervention for weaning in a vulnerable and rapidly maturing population.
47.	Parent–child relationship	Maternal Voice and Infant Sleep in the Neonatal Intensive Care Unit [133]	Renée A Shellhaas et al.	2019	47 neonates	Neonates born at 33–34 weeks did not exhibit increased wakefulness in response to maternal voice, whereas those born at ≥35 weeks’ gestation demonstrated a pronounced response.
48.		Impact of hands-on care on infant sleep in the neonatal intensive care unit [134]	Jennifer Levy et al.	2017	25 infants	Disturbances in sleep and respiration in neonatal intensive care unit (NICU) infants are associated with the frequent hands-on care they receive.
49.		Sleep and salivary cortisol in preterm neonates: a clinical, randomized, controlled, crossover study [135]	Fabrícia Magalhães Araújo et al.	2018	12 neonates	The use of ear protectors in preterm neonates had no impact on salivary cortisol levels or total sleep duration throughout the study periods.

**Table 4:** Caffeine, oxygen, and PSG in infants—a summary of selected studies.

No.	Title	Authors	Year of the Study	Study Population	Conclusions
1.	Effects of caffeine therapy for apnea of prematurity on sleep and neurodevelopment of preterm infants at 6 months of corrected age [136]	Yaprak Ece Yola Atalah et al.	2023	28 infants	Sleep parameters and neurodevelopmental outcomes at 6 months CA showed no differences between infants receiving caffeine therapy and those not.
2.	Caffeine is a respiratory stimulant without effect on sleep in the short-term in late-preterm infants [137]	Maija Seppä-Moilanen et al.	2022	21 infants	The frequency of arousals in response to hypoxia and short-term respiratory activity in late-preterm infants are increased by caffeine.
3.	The effect of caffeine on the ventilatory response to hypercarbia in preterm infants [138]	Thomas Rossor et al.	2018	26 infants	An increase in the ventilatory response to hypercarbia was observed following caffeine administration.
4.	Impact of Supplemental Oxygen on Obstructive Sleep Apnea of Infants [139]	Piyush Das et al.	2018	23 infants	In a sleep lab cohort, low-flow supplemental oxygen effectively treated infant OSA, likely by reducing hypoxemia, airway hypotonia, muscle fatigue, and LG.
5.	Supplemental Oxygen for Treatment of Infants With Obstructive Sleep Apnea [140]	Justin Brockbank et al.	2019	59 infants	A significant reduction in obstructive respiratory events and improved oxygenation, without evidence of impaired alveolar ventilation, was observed in infants with OSA who received supplemental oxygen.
6.	Supplemental Oxygen Treats Periodic Breathing without Effects on Sleep in Late-Preterm Infants [141]	Maija Seppä-Moilanen et al.	2022	18 infants	Periodic breathing and oxygen desaturations were significantly reduced by supplemental oxygen in late-preterm infants, without any alteration in sleep architecture.
7.	Retrospective analysis of inpatient polysomnogram characteristics and discharge outcomes in infants with bronchopulmonary dysplasia requiring home oxygen therapy [142]	Nicole Flores-Fenlon et al.	2021	127 patients	The findings reveal abnormal PSG features in infants with bronchopulmonary dysplasia (BPD) as early as 43 weeks’ CA, not previously reported before initial discharge. Severe BPD was associated with greater respiratory morbidity compared to nonsevere forms at similar CGA.

**Table 5:** Modern methods and devices for monitoring sleep—a summary of selected studies.

No.	Title	Authors	Year of the Study	Study Population	Conclusions
1.	Novel and noninvasive methods for in-home sleep measurement and subsequent state coding in 12-month-old infants [4]	Melissa N. Horger	2022	10 infants	The novel method combining actigraphy and cardiorespiratory monitoring is a feasible, low-resource, caregiver-friendly approach for studying the ultradian cycle, providing high-quality, naturalistic data valuable for infant research.
2.	Actigraphy: Metrics reveal it is not a valid tool for determining sleep in neonates [143]	Matthew Derbin et al.	2022	10 neonates	Without adequate representation of both sleep and wake states, actigraphy cannot be validated for sleep–wake discrimination in preterm infants, as indicated by the findings.
3.	Validation of actigraphy in hospitalised newborn infants using video polysomnography [144]	Mitsuaki Unno et al.	2022	40 newborns	Ankle-mounted actigraphy can provide an accurate assessment of sleep–wake states in neonates hospitalized in the NICU, according to the study.
4.	An Open Source Classifier for Bed Mattress Signal in Infant Sleep Monitoring [45]	Jukka Ranta et al.	2021	43 infants	Signals obtained from a piezoelectric sensor placed beneath the mattress enable technically feasible, automated, noninvasive tracking of sleep state cycles.
5.	Sleep State Trend (SST), a bedside measure of neonatal sleep state fluctuations based on single EEG channels [145]	Saeed Montazeri Moghadam et al.	2022	30 neonates	A single EEG channel allows high-fidelity detection of sleep state fluctuations, which can be presented as a clear and intuitive trend on bedside monitors.
6.	On the development of sleep states in the first weeks of life [146]	Tomasz Wielek et al.	2019	42 infants	Overall, the results demonstrated rapid maturation of newborn sleep characteristics during the first weeks of life, which could be effectively identified using machine learning techniques.
7.	Automated classification of neonatal sleep states using EEG [51]	Ninah Koolen et al.	2017	67 infants	A robust EEG-based sleep state classifier was developed, demonstrating consistent performance across a wide range of postmenstrual ages.
8.	An automated method for coding sleep states in human infants based on respiratory rate variability [56]	Joseph R Isler et al.	2016	49 infants	An automated method using respiratory variability reliably classified infant active sleep (AS) and quiet sleep (QS), matching standard scoring and physiological patterns.
9.	The role of sleep laboratory polygraphy in the evaluation of obstructive sleep apnea syndrome in Robin infants [147]	L. Coutier et al.	2020	20 infants	Given the severity of their condition, polygraphy (PG) appears to be a useful alternative for assessing OSA in infants with PRS, although PSG remains the gold standard for evaluation.
10.	The adapted American Academy of Sleep Medicine sleep scoring criteria in one month old infants: A means to improve comparability? [148]	Anna-Liisa Satomaa et al.	2016	88 infants	The adapted scoring rules demonstrated reproducibility, supporting their application in clinical practice in the absence of standardized recommendations.

## Data Availability

The raw data supporting the conclusions of this article will be made available by the authors on request.

## Abbreviations

AASM	American Academy of Sleep Medicine
AHI	Apnea–hypopnea index
ARI	Acid reflux index
AS	Active sleep
BMI	Body mass index
BMS	Bed mattress sensors
BPD	Bronchopulmonary dysplasia
BVFD	Bilateral vocal fold dysfunction
CAHI	Central apnea–hypopnea index
CAP	Cyclic alternating pattern
CAS	Central apnea syndrome
CHD	Congenital heart disease
CPAP	Continuous positive airway pressure
CRP	C-reactive protein
CSA	Central sleep apnea
CTS	Congenital tracheal stenosis
ECG	Electrocardiogram
EEG	Electroencephalography
ERS	European Respiratory Society
FEMOD	Fast and early mandibular osteodistraction
GER	Gastroesophageal reflux
GERD	Gastroesophageal reflux disease
HR	Heart rate
HRV	Heart rate variability
ICP	Isolated cleft palate
I-DS	Infants with Down syndrome
LG	Loop gain
MDO	Mandibular distraction osteogenesis
NICU	Neonatal intensive care unit
NIV	Non-invasive ventilation
NPV	Negative predictive value
NREM	Non-rapid eye movement
OAI	Obstructive apnea index
OAHI	Obstructive apnea-hypopnea index
OSA	Obstructive sleep apnea
CNS	Central nervous system
QS	Quiet sleep
PB	Periodic breathing
PG	Polygraphy
PMA	Postmenstrual age
PPV	Positive predictive value
PRS	Pierre Robin Sequence
PSG	Polysomnography
REM	Rapid eye movement
SDB	Sleep-disordered breathing
SGP	Supraglottoplasty
SIDS	Sudden infant death syndrome
SpO2	Oxygen saturation
SRBD	Sleep-related breathing disorders
SWA	Slow-wave activity
